# Carob (*Ceratonia siliqua* L.) in Glucose Homeostasis and Energy Balance: The Role of D-Pinitol

**DOI:** 10.3390/nu18091357

**Published:** 2026-04-25

**Authors:** Daniel Torres-Oteros, Emily Pardo-Araujo, Pedro F. Marrero, Sílvia Canudas, Diego Haro, Joana Relat

**Affiliations:** 1 Department of Nutrition, Food Sciences and Gastronomy, School of Pharmacy and Food Sciences, Food Torribera Campus, University of Barcelona, 08921 Santa Coloma de Gramenet, Spain; d.torres.oteros@ub.edu (D.T.-O.); epardoar99@alumnes.ub.edu (E.P.-A.); pedromarrero@ub.edu (P.F.M.); silvia.canudas@ub.edu (S.C.); dharo@ub.edu (D.H.); 2 Institute of Nutrition and Food Safety of the University of Barcelona (INSA-UB), Maria de Maeztu Unit of Excellence, 08921 Santa Coloma de Gramenet, Spain; 3 Centro de Investigación Biomédica en Red de Fisiopatología de la Obesidad y Nutrición (CIBEROBN), Instituto de Salud Carlos III, 28029 Madrid, Spain; 4 Institute of Biomedicine of the University of Barcelona (IBUB), 08028 Barcelona, Spain

**Keywords:** carob, *Ceratonia siliqua*, inositols, D-pinitol, glucose homeostasis, insulin sensitivity, energy balance, functional foods

## Abstract

The global rise in obesity and metabolic disorders has intensified interest in dietary bioactives capable of improving glycemic control and metabolic health. Inositols, particularly D-pinitol, have emerged as insulin-sensitizing cyclitols with potential metabolic relevance. Carob (*Ceratonia siliqua* L.), one of the richest natural sources of D-pinitol, represents a promising nutritional matrix for metabolic regulation. This narrative review critically evaluates current evidence on the role of D-pinitol in glucose homeostasis and energy balance, integrating data from chemical characterization studies, mechanistic research, preclinical models, and human clinical trials assessing purified D-pinitol and D-pinitol–rich preparations, particularly from carob-derived sources. Available evidence suggests that D-pinitol may enhance insulin signaling efficiency, primarily through PI3K/Akt-dependent pathways, modulate hepatic metabolic flexibility, and influence endocrine balance without acting as a classical hypoglycemic agent. Preclinical models consistently report improvements in insulin sensitivity, lipid handling, oxidative stress parameters, and tissue-specific metabolic adaptations. In contrast, clinical trials in healthy, prediabetic, and type 2 diabetic individuals show more heterogeneous outcomes, including attenuation of postprandial glycemia, reductions in circulating insulin and HOMA-IR, and modest improvements in lipid and inflammatory markers. Overall, carob-derived D-pinitol appears to act as a potential insulin-sensitizing metabolic modulator with context-dependent effects influenced by metabolic phenotype and food matrix composition. However, available data remains limited and heterogeneous, with most data derived from preclinical studies and relatively small clinical trials. These findings should therefore be interpreted with caution. Larger, longer-term randomized controlled trials using standardized preparations are required to establish clinical relevance and translational applicability. Notably, the contribution of other bioactive components within the carob matrix cannot be excluded.

## 1. Introduction

The global increase in obesity and associated metabolic disorders, including type 2 diabetes mellitus (T2DM), metabolic dysfunction-associated steatotic liver disease (MASLD), and cardiovascular disease (CVD), represents one of the major public health challenges of the 21st century. These conditions share a common pathophysiological background characterized by chronic low-grade inflammation, oxidative stress, progressive impairment of metabolic homeostasis, immune–metabolic dysregulation, and an acceleration of cellular aging [1,2,3,4,5,6,7,8,9,10].

From a physiological perspective, body weight regulation depends on a dynamic interplay between energy intake, nutrient absorption, metabolic efficiency, neuroendocrine signaling, and energy expenditure. Alterations in any of these pathways can lead to a persistent positive energy balance that drives weight gain and metabolic dysfunction [3,11,12,13]. As a result, increasing attention has been directed toward naturally occurring dietary compounds capable of modulating appetite control, glucose homeostasis, lipid metabolism, and thermogenic/metabolic activity as complementary strategies for weight management and metabolic health [14,15,16,17,18,19].

Within this framework, inositols have emerged as a class of diet-derived bioactive cyclitols involved in the regulation of cellular signaling and metabolic homeostasis [20,21,22,23,24,25,26]. D-pinitol, a naturally occurring methylated derivative of D-chiro-inositol enriched in several leguminous plants, has gained increasing attention as a potential modulator of metabolic health [27,28,29,30]. Carob (*Ceratonia siliqua* L.), one of the richest natural sources identified to date, represents a particularly attractive nutritional matrix for exploring the biological relevance of D-pinitol [28].

This work aims to provide a comprehensive and critical overview of the role of D-pinitol, particularly from carob-derived sources, in the regulation of glucose homeostasis and energy balance. To ensure methodological transparency, a structured literature search was performed using the PubMed database. This narrative review considers in vitro, animal and human studies evaluating the metabolic effects of D-pinitol, as an isolated compound or within enriched preparations and plant-derived matrices, with particular emphasis on carob. Inclusion criteria encompassed studies examining glycemic control, insulin signaling, and lipid metabolism, evaluating both positive and negative findings to provide a balanced synthesis. A formal meta-analysis was not undertaken due to the high heterogeneity of available evidence, particularly regarding formulations and dosing.

## 2. Inositols and D-Pinitol: Chemical and Dietary Aspects

Inositols constitute a family of naturally occurring cyclitols characterized by a cyclohexane ring bearing six hydroxyl groups (C_6_H_12_O_6_). Although they share the same empirical formula as glucose, inositols differ structurally and functionally, acting primarily as signaling molecules, membrane components, and metabolic regulators rather than energy substrates. Nine stereoisomers of inositol have been described, of which myo-inositol is the most abundant biologically active form, while D-chiro-inositol and other isomers occur in smaller amounts in nature [31].

Inositols are present either in free form, as phospholipid components, or as inositol phosphate esters involved in intracellular signaling pathways. Alterations in inositol metabolism have been associated with insulin-resistance-related disorders, including polycystic ovary syndrome, gestational diabetes mellitus, and metabolic syndrome, supporting a link between inositol availability and insulin sensitivity [24,31,32,33]. Numerous experimental and clinical studies have reported beneficial effects of inositol supplementation on insulin sensitivity, glucose homeostasis, and metabolic health [20,23,24,34,35].

As has been mentioned before, D-pinitol (3-O-methyl-D-chiro-inositol) is a naturally occurring methylated derivative of D-chiro-inositol. In this case, the presence of a methoxy group increases chemical stability and may influence D-pinitol absorption and metabolic fate. Furthermore, in vivo, D-pinitol can be demethylated to D-chiro-inositol, which participates in insulin-mediated signal transduction through inositol phosphoglycan second messengers. This mechanism supports its classification as an insulin-mimetic or insulin-sensitizing compound with additional antioxidant, and anti-inflammatory properties [36].

From a nutritional perspective, inositols are widely distributed in plant-based foods, including citrus fruits, whole grains, nuts, seeds, and legumes. However, their concentration and isomer profile vary considerably among food matrices. Legumes are particularly rich in D-pinitol, with soybean, chickpea, lentil, and carob being major dietary sources [37,38,39].

Beyond their dietary distribution, inositols are readily absorbed in the intestine. Myo-inositol is primarily transported through sodium-dependent transporters, whereas D-pinitol and D-chiro-inositol are efficiently absorbed and reach the systemic circulation largely intact, as demonstrated in animal and human pharmacokinetic studies [40,41]. The relatively long half-life reported for D-pinitol further suggests sustained systemic exposure following oral intake [40,41].

## 3. Mechanisms Underlying the Metabolic Effects of D-Pinitol

**Figure 1:**
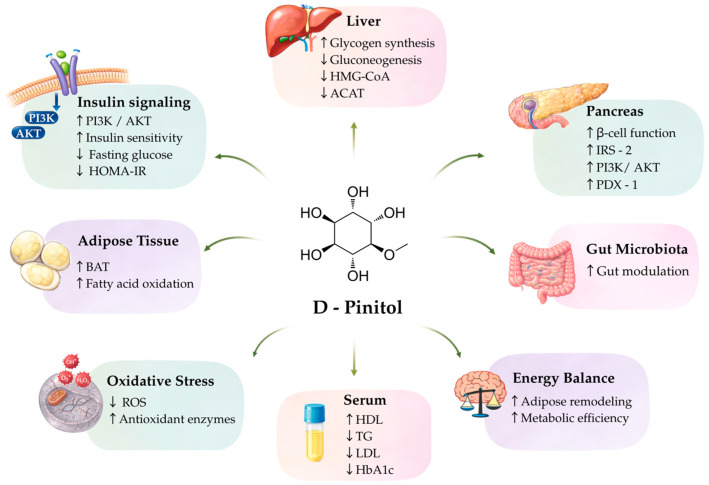
Mechanisms underlying the metabolic effects of D-pinitol. Schematic representation of the molecular and physiological pathways through which D-pinitol may modulate metabolic homeostasis. D-Pinitol influences insulin signaling by enhancing insulin receptor substrate (IRS)-dependent pathways, including phosphoinositide 3-kinase (PI3K)/protein kinase B (AKT) activation, promoting glucose transporter type 4 (GLUT4) translocation and improving insulin sensitivity (HOMA-IR). In the liver, D-pinitol may regulate glycogen synthesis, gluconeogenesis, and lipid metabolism via changes in 3-hydroxy-3-methylglutaryl-coenzyme A (HMG-CoA) and acyl-CoA:cholesterol acyltransferase (ACAT). In the pancreas, it may support β-cell function through insulin receptor substrate 1/2 (IRS-1/2), PI3K/AKT signaling, and pancreatic and duodenal homeobox 1 (PDX-1) regulation. Effects on adipose tissue include stimulation of brown adipose tissue (BAT) activity and increased fatty acid oxidation. D-Pinitol may also reduce oxidative stress by decreasing reactive oxygen species (ROS) and enhancing antioxidant defenses. Modulation of gut microbiota composition and improved systemic metabolic parameters are reflected in changes in serum biomarkers, including triglycerides (TG), high-density lipoprotein (HDL), low-density lipoprotein (LDL), and glycated hemoglobin (HbA1c), contributing to improved energy balance and metabolic efficiency.

Building on its chemical and nutritional characteristics, a growing body of experimental evidence indicates that D-pinitol modulates metabolic homeostasis through multiple targets, including insulin signaling, hepatic carbohydrate metabolism, endocrine regulation, lipid handling, cellular stress responses, and central energy balance pathways (Figure 1).

In this context, it is important to note that most mechanistic insights are derived from in vitro systems and animal models, and their direct translation to human physiology remains limited. Furthermore, variability across experimental models and study designs introduces uncertainty regarding the consistency and generalizability of these findings.

### 3.1. Intracellular Insulin Signaling and Regulation of Hepatic Carbohydrate Flux

D-pinitol has been proposed as an insulin-sensitizing metabolic modulator. Rather than acting as a classical hypoglycemic agent that directly lowers glucose, evidence from both preclinical models and human studies indicate that D-pinitol enhances intracellular insulin signal transduction efficiency, mainly through activation of phosphoinositide 3-kinase (PI3K)/the protein kinase B (AKT) pathway, thereby reproducing key downstream metabolic effects of insulin [20,23,24,33,35].

Experimental studies in cellular systems and animal models of insulin resistance and type 2 diabetes consistently report that chronic D-pinitol administration reduces glycated hemoglobin and pro-inflammatory markers, while it increases plasma insulin and improves fasting glycemia, glucose tolerance, and whole-body insulin sensitivity [36,42]. These metabolic adaptations have been associated with the activation of the PI3K/Akt signaling pathway in tissues such as liver, skeletal muscle, and pancreas [42,43], leading to enhanced glucose transporter 4 (GLUT4) translocation, stimulation of glycogen synthesis, and suppression of hepatic gluconeogenesis, resulting in lower circulating glucose levels and enhanced hepatic glycogen content [43]. Increased activities of hexokinase, pyruvate kinase, and glycogen synthase, together with decreased activities of glucose-6-phosphatase, fructose-1,6-bisphosphatase, glycogen phosphorylase and lactate dehydrogenase have been described in diabetic rodent models treated with purified D-pinitol [42]. Overall, these enzymatic adaptations contribute to improved whole-body metabolic homeostasis and reinforce the concept that D-pinitol primarily optimizes metabolic flux rather than simply lowering circulating glucose levels.

Other studies reported that D-pinitol may indirectly enhance insulin responsiveness by increasing the availability of inositol-derived second messengers or by acting as a metabolic precursor of D-chiro-inositol. Supporting this mechanism, it has been described that, in animal models, chronic administration of purified D-pinitol restores D-chiro-inositol pools and normalizes the D-chiro-inositol/myo-inositol ratio under insulin-resistant conditions [44]. These findings provide a biochemical link between the structural features described in Section 2 and the observed insulin-sensitizing effects.

Within this framework, D-pinitol should be distinguished from other members of the cyclitol family based on its structural topology and metabolic fate [45]. While myo-inositol and D-chiro-inositol primarily act as second messengers for insulin signaling, D-pinitol functions both as a stable dietary precursor to D-chiro-inositol and as an insulin mimetic compound [29,44,46]. Evidence supporting their metabolic effects of myo-inositol and D-chiro-inositol derives from both preclinical models [47,48] and clinical studies [49], consistently reporting improvements in metabolic syndrome-related parameters.

In contrast, the efficacy of D-pinitol appears to be more phenotype-dependent, which may explain the less consistent findings reported [50]. D-pinitol metabolic effects may partly depend on its demethylation to D-chiro-inositol, a process described in humans via the acidic gastric environment or through microbiota-mediated conversion [51]. Notably, its bioavailability may be influenced by the food matrix. For instance, a clinical study in healthy volunteers reported that the presence of monosaccharides in carob syrup reduced D-pinitol absorption by approximately 40%, likely due to competition for intestinal inositol transporters [41]. Together, these factors may contribute to the variability observed in its metabolic effects across studies.

Translational evidence from human intervention studies partially supports these mechanistic observations. In a randomized, double-blind, placebo-controlled trial, administration of D-pinitol reduced fasting glucose, glycated hemoglobin (HbA1c), and Homeostatic Model Assessment of Insulin Resistance (HOMA-IR) without consistent increases in circulating insulin, suggesting improved insulin efficiency rather than enhanced insulin secretion [52]. Similarly, acute clinical intervention studies reported attenuation of postprandial glycemic excursions independent of insulin elevation, reinforcing the concept of peripheral insulin sensitization [53].

### 3.2. Endocrine Modulation, Lipid Metabolism and Oxidative Stress

Apart from its effects on glucose metabolism, D-pinitol modulates endocrine signaling, lipid metabolism and redox balance, indicating a broader role in metabolic homeostasis. Rather than directly stimulating insulin secretion, D-pinitol appears to regulate hormone sensitivity in a context-dependent manner, with responses varying according to metabolic status, treatment duration, and age.

Human and animal studies using purified D-pinitol reveal heterogeneous endocrine outcomes. In human older individuals, acute supplementation is rapidly absorbed but does not consistently improve glucose tolerance, skeletal muscle insulin receptor activation, or insulin-mediated glucose metabolism, indicating modest effects under metabolically normal conditions [54,55]. Similarly, short-term administration in obese subjects with mild type 2 diabetes does not significantly improve insulin sensitivity or endogenous glucose production, suggesting that the metabolic actions of D-pinitol may be more evident in established metabolic dysfunction than in early insulin resistance [56]. In contrast, preclinical studies in diabetic models with β-cell impairment show partial restoration of insulin secretion and improved pancreatic morphology following D-pinitol treatment, underscoring a role in endocrine resilience rather than direct insulinotropic stimulation [46,57].

In parallel with its endocrine effects, D-pinitol also modulates lipid handling and hepatic metabolic flexibility. In diabetic or hyperlipidemic animal models, chronic supplementation with D-pinitol reduces circulating cholesterol, triglycerides (TG), low-density lipoprotein (LDL), and very-low-density lipoprotein (VLDL) fractions, while increasing high-density lipoprotein (HDL), levels, indicating improved lipid homeostasis [58]. Mechanistically, these changes have been associated with decreased cholesterol synthesis in animal studies through inhibition of 3-hydroxy-3-methylglutaryl-coenzyme A (HMG-CoA) reductase and acyl-CoA:cholesterol acyltransferase (ACAT), as well as changes in cytochrome P4502E1 activity [59].

Other studies report suppression of lipogenic pathways such as Sterol receptor element binding protein 1c (SREBP-1c) and increased fatty acid oxidation, reinforcing the role of D-pinitol in regulating hepatic lipid flux [46]. Consistently, in preclinical models of metabolic liver disease, D-pinitol promotes fatty acid oxidation through upregulation of CPT1A [60].

These effects appear to involve additional energy-sensing pathways beyond PI3K/Akt signaling such as AMP-activated protein kinase (AMPK) and mechanistic target of rapamycin (mTOR). Under these conditions, while evidence from myo-inositol and D-chiro-inositol supports regulation of these pathways in both preclinical models [61,62] and clinical settings [22,63], direct evidence for D-pinitol remains limited and, in some cases, inconsistent. In specific models, such as monosodium glutamate (MSG)-obese mice, D-pinitol did not modify glucose homeostasis or circulating lipids but increased hepatic triglyceride content and upregulated genes involved in *de novo* lipogenesis while reducing *Ampk*α expression, highlighting a complex interaction between insulin signaling and hepatic lipid metabolism [46]. These apparently divergent findings highlight a context-dependent effect of D-pinitol on hepatic lipid metabolism, likely influenced by metabolic status and experimental conditions.

In parallel, mTOR signaling may contribute to the metabolic effects of D-pinitol. Acute activation of the PI3K/Akt/mTOR pathway has been reported in the hypothalamus of fasted models, suggesting a potential insulin receptor-independent mechanism in central insulin resistance [64].

Preclinical data also describe adipose tissue remodeling, characterized by reduced white adipose depots and expansion of brown adipose tissue, supporting enhanced energy utilization and metabolic efficiency [59].

Alongside its endocrine and lipid effects, D-pinitol also regulates oxidative stress pathways that contribute to metabolic dysfunction. In streptozotocin-induced diabetic rodent models, chronic treatment attenuates pancreatic oxidative damage, preserves β-cell mass, and enhances antioxidant defenses, highlighting that improved redox balance may contribute to its metabolic benefits [36,57]. Increased antioxidant enzymes activity and reduced reactive oxygen species in hepatic tissue further support a role for D-pinitol in maintaining cellular resilience under metabolic insults [36,59].

### 3.3. Tissue-Specific Actions and Evidence from Non-Carob Plant Sources

Extending the mechanistic framework described above, available data report that D-pinitol exerts tissue-specific actions in pancreatic, intestinal, and cardiovascular systems, reinforcing its role as a multi-target metabolic modulator. Studies using purified D-pinitol and D-pinitol-rich extracts from non-carob sources provide complementary insights into how this cyclitol regulates systemic metabolic homeostasis.

In pancreatic tissue, evidence from in vitro and animal models shows that both purified D-pinitol and D-pinitol-enriched extracts improve islet morphology, enhance β-cell survival, and modulate glucose-stimulated insulin secretion. These effects have been associated with activation of insulin receptor substrate-2 (IRS-2), PI3K/Akt signaling, and the transcription factor PDX-1, supporting a role of D-pinitol in preserving β-cell function under metabolic stress [65,66,67].

In cardiovascular tissue, D-pinitol modulates cellular stress responses and metabolic remodeling. Preclinical studies show reduced accumulation of advanced glycation end-products, suppression of endoplasmic reticulum stress pathways, and regulation of glycophagy signaling in models of diabetic cardiomyopathy, ultimately leading to decreased fibrosis and improved myocardial structure and function [68].

As has been mentioned before, apart from carob, D-pinitol is widely distributed across diverse botanical families, where it functions as an osmoprotectant in response to abiotic or osmotic stress [69]. In the Fabaceae family, soybean (*Glycine max*) represents a major source [69], and clinical trials using soy-derived D-pinitol have demonstrated reductions in postprandial glucose in T2DM patients [53]. Other sources, such as fenugreek (*Trigonella foenumgraecum*) [70,71] and the traditional antidiabetic *Bougainvillea spectabilis* that contains substantial concentrations of this cyclitol [72] have also demonstrated lower glucose-lowering effects in preclinical models. Similarly, extracts from *Mesembryanthemum crystallinum* (ice plant) have shown antidiabetic effects in animal models, including improved glucose tolerance, enhanced β-cell survival, and regulation of insulin secretion [65,66]. D-pinitol has also been identified in the leaves of *Cleistopholis patens*, which has been proposed as a key contribution to its antidiabetic activity in rats [66].

In this context, evidence derived from plant extracts also underscores that D-pinitol may influence gut–metabolic interactions. In diabetic animal models, administration *Mesembryanthemum crystallinum* extracts improve glucose tolerance and modulate gut microbiota composition, suggesting that intestinal signaling pathways may contribute to D-pinitol whole-body metabolic regulation [66]. These effects may involve changes in short-chain fatty acid production and gut–liver axis signaling; however, current evidence is scarce, largely indirect, and primarily derived from preclinical models.

Despite its presence in multiple species, translating evidence from non-carob sources remains challenging. Most studies rely on whole extracts rather than purified compounds, making it difficult to isolate the specific contribution of D-pinitol from other coexisting bioactive components, such as dietary fiber and polyphenols, which are known to independently influence metabolic and gut-related pathways, in both human and animal models [73,74]. Microbiota-mediated processes, including changes in enzyme activities and the production of specific metabolites may further contribute to the observed effects [75,76]. Nevertheless, the number of available experimental models remains relatively small, which may restrict the generalizability of these findings, particularly to human physiology.

Overall, D-pinitol emerges as a context-dependent metabolic modulator rather than a classical hypoglycemic agent. Its actions extend from glucose regulation to coordinated effects on insulin signaling, lipid metabolism, endocrine balance, and redox pathways across multiple tissues. Importantly, its responses vary according to metabolic status and tissue context, supporting a role in improving metabolic efficiency and cellular resilience under chronic metabolic stress. This mechanistic framework provides the basis for evaluating D-pinitol within complex food matrices such as carob and its potential relevance in nutritional and clinical settings.

## 4. Carob as a Rich Source of D-Pinitol

Carob (*Ceratonia siliqua* L.) is an evergreen tree belonging to the Leguminosae family, traditionally cultivated in arid and semi-arid areas of Mediterranean regions and increasingly valued for its nutritional and functional properties. Carob pods are edible beans composed of pulp (approximately 90%) and seeds (approximately 10%). While seeds are mainly used for galactomannan gum production, the pulp is the primary source of soluble sugars, dietary fiber, polyphenols, and inositols [38,39,77,78,79].

Carob pulp contains 45–56% in sugars (*w*/*w*), predominantly sucrose (65–75% of total sugars) with smaller amounts of glucose (2–4%) and fructose (6–7%). Minor oligosaccharides including maltose, raffinose, raffinose, stachyose, verbascose, and xylose, further contribute to its distinctive nutritional profile [39,77,80]. In addition to carbohydrates, carob pulp is also characterized by a high cyclitol content [38,77,78].

Carob pods represent one of the richest known dietary sources of D-pinitol, reaching 5–7.5% of dry weight in carob pod powder and exceeding levels reported in soybean, chickpea, lentil, and other commonly consumed legumes [28,37,38,39,81]. Together with D-pinitol, carob contains other cyclitols, including myo-inositol (approximately 0.5–1% of dry weight) and D-chiro-inositol (around 0.1%), providing a mixture of stereoisomers that may exert additive or synergistic effects. Chromatographic and mass spectrometric analyses have identified multiple inositols and related polyols in carob pulp and derived products [28,81,82].

Carob composition and final cyclitol profile vary depending on genotype, growing conditions, and processing [83]. Aqueous extraction and concentration, commonly used in syrup production, enrich water-soluble compounds such as D-pinitol, resulting in higher concentrations than in raw pods [40,60,84,85,86]. This makes carob syrups particularly efficient vehicles for delivering physiologically relevant amounts of D-pinitol within realistic serving sizes.

Despite these advances, key food chemistry aspects remain insufficiently characterized, particularly regarding the stability of D-pinitol under different processing conditions, as well as its bioaccessibility and bioavailability within complex food matrices. These factors may significantly influence its biological activity and should be considered when interpreting findings.

Beyond inositols, carob pods provide up to 40% dietary fiber [39], with the insoluble fraction mainly composed of cellulose, hemicellulose and lignin [80], along with substantial amounts of phenolic compounds, including phenolic acids, gallotannins, and flavonoids [84,87,88,89], many of which are partially bound to the fiber fraction [84,87,88,89,90,91,92,93]. The coexistence of these components suggests that the metabolic effects of carob consumption reflect combined bioactive actions. Dietary fiber and polyphenols have independently been associated with improvements in glycemic control, delayed carbohydrate absorption, modulation of gut microbiota, and reduced postprandial glucose excursions [90]. Accordingly, the metabolic effects observed in carob powder-based interventions likely result from the combined action of D-pinitol and these additional bioactive compounds. In contrast to carob powders that retain a complete matrix of fiber and polyphenols, liquid formulations such as syrups and beverages typically undergo filtration and chromatographic separation that remove part of other bioactive compounds while enriching D-pinitol within a simplified profile, allowing a more targeted assessment of its specific metabolic effects [60,81,86,94].

Consistently, carob-based products have shown beneficial effects in obesity, dyslipidemia, insulin resistance, and metabolic dysfunction [77,78,90,95,96,97], supporting its investigation as a functional matrix for modulating glucose homeostasis and energy balance.

## 5. Preclinical Evidence from Carob-Derived D-Pinitol and Pinitol-Rich Matrices

Building on the compositional framework described in Section 4, preclinical studies provide translational insight into the metabolic effects of D-pinitol delivered either as a purified compound or within carob-derived preparations. In rodent models of insulin resistance, diabetes, obesity, and dyslipidemia, both forms impact in endocrine signaling, hepatic metabolism, lipid handling, and tissue-specific glucose regulation, confirming that D-pinitol remains biologically active within the carob-derived matrices (Table 1).

The available preclinical evidence remains heterogeneous in terms of experimental models, dosages, intervention duration and formulations, which limits direct comparability across the studies. Interpretation is further constrained by the compositional complexity of these preparations, where the specific contribution of D-pinitol cannot always be clearly distinguished from that of coexisting bioactive compounds such as fiber and polyphenols.

Pharmacokinetic studies using carob-derived purified D-pinitol formulations have shown rapid intestinal absorption and systemic distribution after oral administration. In fasted Wistar rats, D-pinitol appears in plasma within minutes, accumulates in metabolically active tissues such as the liver, and remains detectable for several hours, indicating a relatively long half-life and sustained exposure without hepatic or renal toxicity [98]. Moreover, acute administration has been associated with reduced circulating insulin and HOMA-IR, together with increased ghrelin levels and a higher glucagon/insulin ratio while glycemia remains largely unchanged. This endocrine profile aligns with improved metabolic efficiency rather than direct hypoglycemic action [64,98]. Mechanistically, these effects have been related to the downregulation of hepatic pyruvate kinase expression [98] with no significant changes in the mRNA levels of gluconeogenic genes and activation of hypothalamic PI3K/Akt signaling [64], supporting both peripheral and neuroendocrine mechanisms.

On the other hand, carob pod-derived preparations standardized for D-pinitol content have shown comparable metabolic effects in rodent models under both subchronic and chronic interventions. Administration of a D-pinitol-enriched carob syrup (InnoSweet^®^), obtained by aqueous extraction, improves fasting and postprandial glycemic responses, enhances glucose tolerance, and increases circulating ghrelin levels, closely reproducing the endocrine profile observed with equivalent doses of purified D-pinitol [60]. In these conditions, prolonged intake further reduces hepatic lipid accumulation, lowers liver glycogen content, and promotes metabolic adaptations characterized by increased fatty acid oxidation and coordinated regulation of glycolytic and gluconeogenic pathways, without detectable toxicity [60].

Similarly, D-pinitol-enriched carob beverages improve metabolic outcomes in models of metabolic dysfunction. In Zucker diabetic fatty (ZDF) rats, chronic intake of a low-glycemic index carob preparation prevents the progressive rise in fasting glucose observed in sucrose-treated controls, while increasing circulating complement component C4A and upregulating jejunal *Glucose transporter 2* (*Glut2*) expression [85]. These adaptations suggest that carob-derived D-pinitol modulates intestinal glucose transport and pancreatic endocrine function, contributing to improved systemic glucose homeostasis.

Importantly, not all experimental studies report uniformly beneficial metabolic outcomes, indicating that D-pinitol effects may be dependent on the metabolic context and the experimental conditions (Table 1).

Overall, preclinical data supports the biological activity of D-pinitol within carob-derived matrices and highlights its capacity to modulate multiple metabolic pathways, including insulin secretion, central neuroendocrine signaling, hepatic carbohydrate and lipid metabolism, and intestinal glucose handling, in both acute and chronic approaches.

From a translational perspective, these observations connect molecular mechanisms described in Section 3 with the emerging clinical evidence described below. The consistent metabolic improvements described across rodent models provide a strong biological rationale for human intervention studies and support the potential of carob-derived D-pinitol formulations as a nutritionally relevant strategy to modulate glucose homeostasis and metabolic health.

## 6. Human Evidence on the Metabolic Effects of D-Pinitol in Carob-Derived Preparations

Consistent with the mechanistic and preclinical evidence summarized above, human intervention studies provide critical insight into the metabolic effects of D-pinitol delivered within carob-derived matrices. Across healthy individuals, subjects with impaired fasting glucose, and patients with type 2 diabetes, clinical trials consistently show that carob-derived D-pinitol modulates endocrine responses, glycemic variability, and cardiometabolic risk (Table 2).

Despite these promising findings, the available clinical evidence remains heterogenous. Differences in study design, including dosage, formulations, intervention duration and participant characteristics, complicate direct comparisons and dose–response interpretation.

Initial trials using purified D-pinitol demonstrated that a single oral dose in healthy volunteers reduces circulating insulin and attenuates postprandial hyperglycemia without inducing hypoglycemia, supporting improved peripheral glucose handling and metabolic efficiency rather than increased insulin secretion [40]. This endocrine signature closely resembles the adaptations reported in preclinical models and provides early clinical validation for the insulin-sensitizing mechanisms described previously.

Nevertheless, these effects have not been consistently reproduced across all populations, particularly in metabolically healthy individuals, where improvements in insulin sensitivity tend to be modest or absent [29].

Subsequent randomized controlled trials have primarily evaluated standardized carob-derived preparations, including the D-pinitol-enriched beverage Fruit Up^®^ and the carob syrup InnoSweet^®^. While both products deliver high concentrations of D-pinitol, they differ significantly in their phytochemical composition. Fruit Up^®^ is a complex food matrix rich in polyphenols and soluble fiber, specifically arabinoxylans [99]. In contrast, InnoSweet^®^ is a more refined preparation obtained through filtration and chromatographic processing, which maintains a balanced sugar-to-pinitol ratio while removing most insoluble fibers and complex phytochemicals, resulting in a simpler sweetener profile [60]. As a result, in more complex formulations, synergistic interactions may contribute to the observed metabolic responses, making it difficult to attribute effects exclusively to D-pinitol.

Chronic supplementation with Fruit Up^®^ (4 g/day of D-pinitol) has been associated with improvements in insulin sensitivity, reduced circulating insulin and HOMA-IR, and attenuated postprandial glucose excursions in normoglycemic, impaired fasting glucose (prediabetic), and diabetic individuals [99,100,101]. Moreover, continuous glucose monitoring confirmed reduced daily glycemic variability [99]. In this context, even global metabolic outcomes were similar, stratified analyses indicated phenotype-dependent responses.

In healthy individuals, improvements were more evident in lipid-related biomarkers, including reductions in ApoB and increases in LDL particle size, suggesting an overall improvement in cardiometabolic risk [99]. In contrast, in prediabetic cohorts with impaired fasting glucose, supplementation with Fruit Up^®^ resulted in differential responses according to adiposity status and body mass index [100]. Then, while in non-obese subjects, the intake of the beverage resulted in attenuated postprandial and nocturnal glucose excursions with no changes in inflammatory cytokines, obese participants exhibited smaller overnight glycemic fluctuations accompanied by a significant decrease in inflammatory markers, including IL-6 and TNF-α, potentially mediated through unfolded protein response and sirtuin 1 signaling [100,102].

These observations point out that the metabolic response to carob-derived D-pinitol is highly influenced by baseline metabolic status, adiposity and inflammatory profile, thus underscoring a phenotype-specific effect where improvements in insulin sensitivity predominate in non-obese individuals, whereas anti-inflammatory responses appear more pronounced in obese subjects, which may partly explain the variability observed across clinical studies.

Finally, in patients with established type 2 diabetes, chronic consumption of the inositol-enriched beverage improved glycemic control, lowered TG and HbA1c levels, enhanced endothelial function, and reduced oxidative stress markers, indicating pleiotropic benefits extending to vascular health [101]. Nevertheless, the magnitude of these effects varies across studies, and the relatively small sample sizes and short intervention periods limit the generalizability of these findings.

On the other hand, short-term interventions using the same pinitol-enriched beverage have reported increased circulating levels of insulin-like growth factor acid-labile subunit (IGF1BP-ALS) and the complementary component C4A in individuals with impaired glucose tolerance but not in healthy volunteers [100]. Notably, elevations in C4A have also been reported in ZDF rats, reinforcing the translational consistency between preclinical and clinical data [85].

Clinical trials have also evaluated a standardized carob syrup (InnoSweet^®^) delivering D-pinitol within a syrup-based matrix. In a randomized crossover study in healthy volunteers, ingestion of InnoSweet^®^ produced a less sustained glycemic excursion, a lower insulin response to induced hyperglycemia, and a higher glucagon/insulin ratio compared with glucose at an equivalent carbohydrate load, supporting a modulatory effect of the carob matrix on endocrine and glycemic responses [60].

Complementary pharmacokinetic analyses revealed rapid intestinal absorption of D-pinitol with prolonged systemic persistence; importantly, co-administration within carbohydrate-rich carob matrices appeared to modify absorption kinetics, indicating that the food matrix influences bioavailability and endocrine effects [41]. Moreover, administration of purified D-pinitol also produced mild endocrine changes, including small variations in circulating insulin, glucagon, and ghrelin, while plasma glucose remained largely unaffected [41]. These findings further support the notion that D-pinitol acts as a metabolic modulator rather than a direct hypoglycemic agent, although the clinical relevance of these endocrine changes remain to be fully established.

Recently, two complementary randomized controlled trials assessed the metabolic impact of commercially available carob syrups on glycemic response, lipid profile, and anthropometric parameters in healthy adults [94]. Both syrups showed low glycemic index values (~56–60%) and significantly reduced postprandial glucose responses compared with a glucose reference intake, indicating a lower glycemic load despite their high sugar content. A six-week daily supplementation led to modest but significant reductions in total cholesterol and waist circumference, along with favorable trends in TG, visceral fat, and body composition. These effects may be attributed to bioactive compounds in the carob syrup; in contrast, the magnitude of metabolic improvements was modest, likely due to the normoglycemic and normolipidemic status of participants and the short intervention period [94].

Finally, a randomized crossover trial evaluated the acute postprandial effects of an Imera cultivar carob beverage in healthy adults. Data presented indicated that consumption of carob powder significantly reduced early postprandial glucose and insulin excursions compared with an isosaccharinic sucrose control, particularly within the first 60 min after ingestion [103]. The LC–MS analysis identified a diverse phenolic profile, D-pinitol and insoluble fiber fractions in the beverage, which may contribute to glycemic modulation through inhibition of carbohydrate-digesting enzymes, antioxidant activity, and enhancement of insulin signaling. In this case, although total incremental AUC over 180 min did not differ significantly, early metabolic responses were attenuated, indicating a potential role of carob in postprandial glycemic regulation [103].

Taken together, available clinical data suggest that D-pinitol, particularly when delivered within carob-derived matrices, may contribute to improvements in glycemic control and metabolic health. However, the overall evidence is constrained by heterogeneity in study design, variability in formulations, and population-specific responses. Well-designed, long-term randomized controlled trials using standardized formulations and clearly defined metabolic phenotypes are required to establish the clinical efficacy, safety, and translational relevance of D-pinitol consumption. Importantly, most clinical evidence derives from studies using complex carob-derived preparations, where the contribution of D-pinitol cannot be fully distinguished from that of other bioactive components.

**Table 2:** Human intervention studies evaluating the metabolic effects of purified D-pinitol and D-pinitol-rich carob preparations. Low-density lipoprotein (LDL); Insulin-like growth factor acid-labile subunit (IGF1BP-ALS); Complement component (C4A); Reactive oxygen species (ROS).

Ref.	D-Pinitol Source	Experimental Model	Dose/Sample Size	Exposure Design	Metabolic Outcomes
[40]	Fruit Up^®^	Human randomized parallel trial with a 3-arm design single-blinded placebo controlled and cross-over. Healthy individuals	2.5, 4.0 or 6.0 g of pinitol (n = 10/group)	Oral intake Acute	↓ Postprandial glucose and insulin
[99]	Fruit Up^®^	Human randomized controlled-placebo parallel trial with a 3-arm design. Healthy/IGF/T2D individuals	4 g/day of pinitol (n = 40/group)	Oral intake 12 weeks	↓ Insulin, ↓ Glycemia ↓ HOMA-IR ↓ ApoB ↑ LDL size
[100]	Fruit Up^®^	Human double-blind, randomized, controlled trial. Prediabetic individuals	4 g/day of pinitol IEB (n = 24)	Oral intake 12 weeks	Non-obese: ↓ Insulin; ↓ HOMA-IR Obese: ↓ Glycemia after overnight fasting ↓ IL-6 and TNF-α
[85]	Fruit Up^®^	Human double-blind, randomized, controlled trial. Healthy and prediabetic individuals	4 g/day of pinitol (n = 20/group)	Oral intake 6 weeks	↓ Glycemia Prediabetic: ↓ IGF1BP-ALS ↓ C4A
[101]	Fruit Up^®^	Human randomized, double-blind, controlled study. Type 2 diabetes individuals	4 g/day of pinitol (n = 19/group)	Oral intake 12 weeks	↓ Glycemia ↓ Triglycerides ↓ HbA1c levels ↑ Endothelial function ↓ ROS
[60]	InnoSweet^®^	Human randomized trial. Healthy individuals	1.6 g of pinitol (n = 9)	Oral intake Acute	↓ Glycemic excursion ↑ Glucagon/insulin ratio
[41]	Pure pinitol vs. InnoSweet^®^	Human randomized trial. Healthy individuals	D-Pinitol 5 or 15 mg/Kg BW (n = 6; n = 10), 1.6 g of pinitol from carob syrup (n = 9)	Orally Acute	High dose of D-pinitol: ↓ Insulin ↑ Glucagon and ghrelin
[94]	Carob Syrup Ceratonia + (Black Essence^®^ vs. Gold Essence^®^)	Human randomized and controlled trials. Healthy individuals	15.7 g of Syrup + Black Essence^®^ or 15.7 g of Carob + Gold Essence^®^ diluted in 250 mL of water (0.94 g and 1.57 g D-pinitol) (n = 20/group)	Oral intake Acute	↓ Glycemia
			10 g/day of Ceratonia + Black Essence^®^ carob syrup with D-pinitol (1.0 g of pinitol) (n = 37)	Oral intake 6 weeks	↓ Glycemia ↓ Total cholesterol ↓ Waist circumference
[103]	Carob powder beverage	Human acute randomized controlled, crossover trial. Healthy male individuals	30 g of carob powder (n = 15)	Orally Acute	↓ Postprandial glycemia

## 7. Concluding Remarks

The evidence summarized in this review positions carob-derived D-pinitol as a context-dependent metabolic modulator rather than a classical hypoglycemic compound. Across mechanistic, preclinical, and clinical studies, D-pinitol has been associated with improvements in insulin signaling and coordinated metabolic regulation, suggesting that its effects rely on improving intracellular signaling efficiency and metabolic flexibility rather than directly lowering circulating glucose.

Importantly, D-pinitol also exerts tissue-specific actions including pancreatic β-cell preservation, hepatic metabolic regulation, adipose tissue remodeling, intestinal glucose handling, and cardiovascular stress responses. This multi-target profile supports a system-level mechanism integrating endocrine resilience, nutrient sensing, and redox homeostasis.

At this point it is critical to distinguish between established mechanisms and preliminary hypotheses. While the PI3K/Akt pathway is well-documented, other proposed effects such as the activation of brown adipose tissue (BAT) or specific shifts in gut microbiota composition remain preliminary hypotheses requiring further validation.

Notably, D-pinitol effects appear to depend strongly on baseline metabolic status, with more consistent benefits observed under metabolic dysfunction, whereas normometabolic states typically show modest or subtle responses, highlighting a phenotype-dependent action.

The contribution of coexisting bioactive components within the carob-derived preparations cannot be clearly separated based on available data. Nevertheless, the convergence of results from purified D-pinitol and standardized carob-derived preparations supports the concept of carob as a functional delivery matrix rather than simply a sugar-rich product. The metabolic efficacy of carob-based interventions appears to depend on the complexity of the formulation employed: while powders and certain beverages retain a broad range of fibers and polyphenols that may act synergistically, more refined preparations provide simplified profiles that allow a clearer assessment of D-pinitol specific effects.

Clinical trials consistently report reductions in circulating insulin and HOMA-IR, attenuation of postprandial glycemic excursions, and modest improvements in lipid and inflammatory markers. However, the available human evidence remains constrained by small sample sizes, short intervention periods, heterogeneity in study design and variability in formulations and dosing strategies (see Section 7.1). In addition, most studies have been conducted in relatively small cohorts of healthy or moderately dysmetabolic individuals, restricting generalizability to broader clinical populations. Overall, inconsistencies across studies and population-specific responses underscore the need for cautious interpretation of the current evidence.

### 7.1. Limitations of the Available Data

The available literature presents several important limitations that should be considered when interpreting these findings.

First, a substantial proportion of the mechanistic and metabolic evidence derives from in vitro and animal models, which may not fully translate to human physiology. Second, clinical studies remain limited in number and are characterized by small sample sizes, short intervention durations, and considerable heterogeneity in study design, formulations, and dosing strategies. Third, the complexity of the carob matrix makes it difficult to clearly separate the specific contribution of D-pinitol from that of other coexisting bioactive compounds, such as dietary fiber and polyphenols. Finally, the lack of standardized preparations and consistent methodological approaches further complicates direct comparisons across studies and the establishment of dose–response relationships.

### 7.2. Future Research

From a translational perspective, the reproducibility of metabolic improvements across experimental models provides a solid mechanistic rationale for further human research.

Future studies should prioritize well-designed, large-scale, long-term randomized controlled trials using well-characterized and standardized carob-derived preparations, together with stratification by metabolic phenotype. Particular attention should be given to the establishment of dose–response relationships, the identification of optimal formulations, and the evaluation of long-term safety.

Importantly, future research should also address current gaps by including diverse and vulnerable populations, such as individuals with advanced metabolic disease, elderly subjects, and other underrepresented groups. The integration of mechanistic biomarkers, continuous glucose monitoring, and multi-omics approaches may help clarify interindividual variability and matrix–bioactive interactions.

In addition, advancing the translational potential of D-pinitol will also require the development of standardized dosing strategies, the evaluation of combination approaches with existing therapies, and the implementation of personalized nutritional interventions tailored to metabolic phenotype.

In summary, carob-derived D-pinitol emerges as a promising insulin-sensitizing cyclitol with pleiotropic metabolic effects that extend beyond glucose regulation to lipid metabolism, inflammatory balance, and tissue resilience. However, although current evidence supports its potential as a functional nutritional strategy in metabolic health, definitive clinical validation remains limited and requires further rigorous investigation.

## Figures and Tables

**Table 1:** Preclinical translational evidence on the metabolic effects of purified and carob-derived D-pinitol across experimental models. Homeostatic Model Assessment of Insulin Resistance (HOMA-IR); Phosphoinositide 3-kinase (PI3K)/protein kinase B (AKT); Insulin-glucagon ratio (IGR).

Ref.	D-Pinitol Source	Experimental Model	Dose/Sample Size	Exposure Design	Metabolic Outcomes
[98]	Purified D-pinitol 98% (Caromax^®^)	Fasted Wistar rats	100 or 500 mg/kg BW (n = 5; n = 8)	Acute Oral gavage	↓ Insulin; ↓ HOMA-IR; ↑ Ghrelin; ↑ Insulin tolerance; = Glycemia; ↓ Liver pyruvate kinase expression
[64]	Purified D-pinitol 98% (Caromax^®^)	Wistar rat	500 mg/kg BW (n = 8)	Acute Oral gavage	↓ Insulin; ↓ HOMA-IR; ↓ IGR; ↑ Pl3K/AKT signaling in hypothalamus
[60]	D-pinitol in a standardized carob syrup (InnoSweet^®^)	Wistar rat	100 mg/kg BW (n = 10)	Acute Oral gavage	↓ Glucose
				Subchronic (10 days) Drinking water	↑ Glucose tolerance; ↑ Ghrelin; ↓ Liver fat and glycogen ↑ Liver fat oxidation, gluconeogenesis and glycolytic pathways
[85]	Carob-pod, pinitol-enriched beverage (PEB; Fruit Up^®^)	Male Zucker diabetic fatty (ZDF) rats	1.15 g/kg BW/day (n = 5)	Chronic (28 days) Oral gavage	↓ Glucose level change ↑ Serum C4A ↑ Jejunum *Glut2* expression

## Data Availability

No new data were created or analyzed in this study. Data sharing is not applicable to this article.
